# Identification and characterization of two zebrafish Twik related potassium channels, Kcnk2a and Kcnk2b

**DOI:** 10.1038/s41598-018-33664-9

**Published:** 2018-10-17

**Authors:** Nathalie Nasr, Adèle Faucherre, Marc Borsotto, Catherine Heurteaux, Jean Mazella, Chris Jopling, Hamid Moha ou Maati

**Affiliations:** 1 IGF, CNRS, INSERM, Université de Montpellier, Labex ICST, F-34094 Montpellier, France; 2 IPMC, CNRS, INSERM, Université de Nice Sophia Antipolis, Labex ICST, F-06560 Valbonne, France

## Abstract

KCNK2 is a 2 pore domain potassium channel involved in maintaining cellular membrane resting potentials. Although KCNK2 is regarded as a mechanosensitive ion channel, it can also be gated chemically. Previous research indicates that *KCNK2* expression is particularly enriched in neuronal and cardiac tissues. In this respect, KCNK2 plays an important role in neuroprotection and has also been linked to cardiac arrhythmias. KCNK2 has subsequently become an attractive pharmacologic target for developing preventative/curative strategies for neuro/cardio pathophysiological conditions. Zebrafish represent an important *in vivo* model for rapidly analysing pharmacological compounds. We therefore sought to identify and characterise zebrafish *kcnk2* to allow this model system to be incorporated into therapeutic research. Our data indicates that zebrafish possess two *kcnk2* orthologs, *kcnk2a* and *kcnk2b*. Electrophysiological analysis of both zebrafish Kcnk2 orthologs shows that, like their human counterparts, they are activated by different physiological stimuli such as mechanical stretch, polyunsaturated fatty acids and intracellular acidification. Furthermore, both zebrafish Kcnk2 channels are inhibited by the human KCNK2 inhibitory peptide spadin. Taken together, our results demonstrate that both Kcnk2a and Kcnk2b share similar biophysiological and pharmacological properties to human KCNK2 and indicate that the zebrafish will be a useful model for developing KCNK2 targeting strategies.

## Introduction

The two pore domain (K_2_P) channels are the most recent addition to the large family of potassium channels. They consist of two pore-forming P loops and four transmembrane segments^1^. These channels can be found in several excitable and non-excitable cell types where they play a role in maintaining the membrane resting potential^1,2^. The K_2_P potassium channel family is made up of fifteen members, divided into separate subfamilies based on their expression pattern, function and electro/biophysical properties^1,2^. Members of the K_2_P family perform a diverse range of physiological roles and have been associated with a variety of pathologies. For example, a missense mutation in *KCNK9* causes Birk Barel mental retardation syndrome^3^, while a dominant negative mutation in *KCNK18* has been linked to familial migraine^4^. In mice, deletion of both *KCNK3* and *KCNK9* leads to primary hyperaldosteronism syndrome^5^, while variants in *KCNK3* have been associated with this condition in humans^6^. In zebrafish, Kcnk1 has a role in the regulation of heart rate and atrial size^7^. Among the K_2_P family, KCNK2 has been the subject of extensive research^1,2^. KCNK2 channel activity is polymodally regulated and as such a range of endogenous physiological stimuli can modulate its activity. For example, both mechanical membrane stretch and decreased intracellular pH promote KCNK2 activity^8,9^. KCNK2 is also sensitive to volatile anaesthetics such as chloroform, halothane, isoflurane and desflurane as well as other gases and gaseous compounds such as xenon, cyclopropane and nitric oxide^10^. Furthermore, polyunsaturated fatty acids (PUFAs) such as arachidonic acid (AA), docosahexaenoic acid (DHA) and alpha-Linolenic acid (ALA) along with lysophospholipids (LPL) such as lysophosphatidylcholine (LPC) are all capable of activating KCNK2^9,11–14^. GPCRs are also able to both positively and negatively modulating KCNK2 activity, a process reliant on the phosphorylation of critical serine residues by either PKA or PKC^15–18^. Pharmacologically, KCNK2 is insensitive to all of the classical potassium channel inhibitors such as TEA (tetraethylamonium) and 4-AP (4-aminopyridine), which block voltage gated potassium channels, and glibenclamide, apamine and charybdotoxine which inhibit ATP and calcium sensitive potassium channels^19^. However, this channel is sensitive to antidepressant selective serotonin reuptake inhibitors (SSRIs) such as fluoxetine, and next generation antidepressants such as the small peptide spadin, all of which effectively inhibit its activity^9,15–17,20–24^. Lastly, riluzole, a neuroprotective molecule, can both activate and inhibit KCNK2^9,25^. Activation is accomplished by a direct interaction between KCNK2 and riluzole, while the inhibitory effect is indirect and mediated by PKA phosphorylation of KCNK2. Due to its polymodal activation/inhibition and expression in a variety of biological tissues, KCNK2 is involved in a broad range of physiological and pathophysiological processes^13,15,16,22,23,26–30^. *KCNK2* is highly expressed in the central nervous system (CNS) and as such has been linked to a variety of neuropatholgies such as depression, pain and stroke^31–33^. Indeed, *Kcnk2* knockout mice show a resistance to depression due to enhanced 5-hydroxytryptamine (serotonin) neurotransmission and an increase in neurogenesis^15^. As such KCNK2 has becoming a highly attractive target for treating this condition in humans. A pronounced expression of *Kcnk2* has also been observed in sensory neurons where it is required for polymodal nociception. Consequently, disrupting *Kcnk2* in mice makes them more sensitive to painful thermal and mechanical stimuli^34^. KCNK2 has also been shown to play an important role in neuroprotection against epilepsy and stroke. In particular, mice which lack a functional KCNK2 show an increased sensitivity to these pathologies. Mechanistically, it appears that KCNK2 mediates the beneficial neuroprotection provided by PUFAs and LPLs^29^. In the heart, KCNK2 is a key component of mechano-electric coupling and is able to modulate the ventricular action potential with an important role in the repolarization of the membrane potential^35,36^. In cardiac tissue, KCNK2 expression is regulated by the POPEYE domain proteins such as POPDC and POPDC2. Deletion of these proteins in mice induces an age and stress dependant sino-atrial bradycardia^37^. Moreover, an atrio-ventricular block has been observed in double POPDC1 and 2 knock out mice as well as in POPDC2 knock down zebrafish^38,39^. Consequently in humans, mutations in *POPDC1* are responsible for atrio-ventricular block due to dysregulated KCNK2 activity^35^. Other recent studies have shown that aberrant *KCNK2* expression is also associated with physiopathological cardiac conditions. Indeed, increased *KCNK2* expression has been observed in patients suffering from pathological ventricular hypertrophy while decreased expression has been linked to atrial fibrillation after cardiac remodelling^40,41^.

The zebrafish is rapidly becoming an established *in vivo* model system for addressing a wide variety of physiological and pathophysiological situations. Indeed, assays have been developed which cover a wide range of conditions associated with KCNK2, ranging from depression and nociception to cardiac form and function. Because of the potential therapeutic applications associated with KCNK2, we have endeavoured to characterise this gene in zebrafish to imitate a novel *in vivo* model for developing KCNK2 targeted therapeutics.

## Results

### Zebrafish posess two *KCNK2* orthologs

Analysis of the Ensembl database indicates that the zebrafish posess 2 orthologues of *KCNK2* (*kcnk2a* ENSDARG00000055123 and *kcnk2b* ENSDARG00000007151 respectively); this is most likely the result of an ancient genome duplication. To study their biophysical properties, we first cloned both orthologs from a three days post fertilization (dpf) embryonic zebrafish cDNA library. Sequence analysis indicates that both genes are highly homolgous to human *KCNK2* (*kcnk2a-*75.7%, *kcnk2b-*72.8%) (Suppl. Fig. 1). Because both *kcnk2* orthologs show a high homology to the human *KCNK2* gene, this indicates that the zebrafish represents a potentially useful *in vivo* model for developing pharmacological KCNK2 targeted therapeutics. Next we sought to determine where both zebrafish *KCNK2* ortholgues are expressed during embryonic development. To achieve, this we performed *in situ* hybridisation on 4dpf zebrafish embryos using antisense RNA probes synthesised from either *kcnk2a* or *kcnk2b*. In this manner we were able to determine that both genes are highly expressed in the developing zebrafish brain as has been reported in mammals (Suppl. Fig. 2A,B).

### Kcnk2a and Kcnk2b are activated by mechanical force

**Figure 1:**
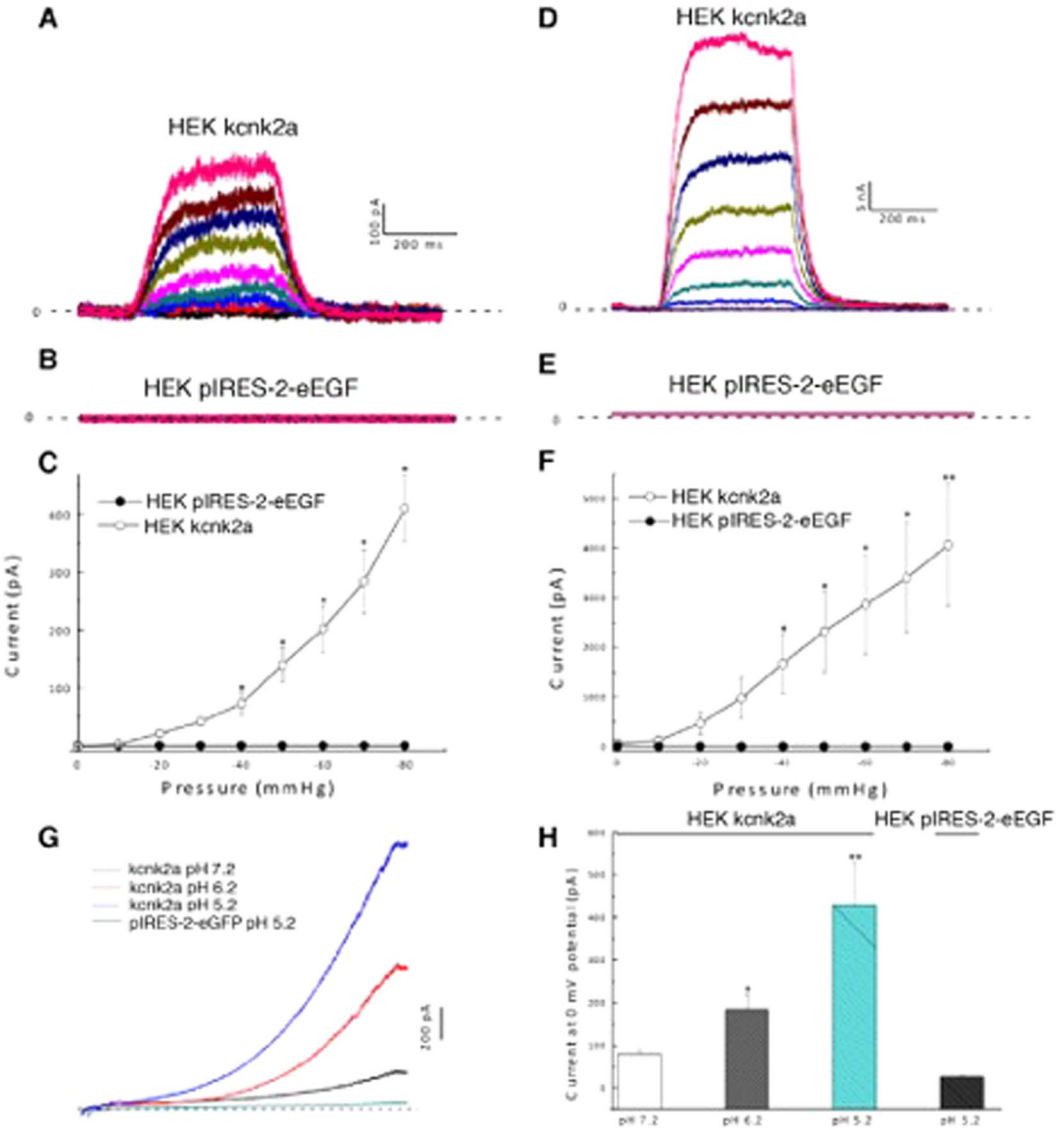
(**A–C**) Recorded currents at 0 mV potential by applying an increased negative pressure from 0 to −80 mmHg on HEK cells transfected with kcnk2a-pIRES-2-eGFP in CA configuration (n = 3) (**A**) on HEK cells transfected with pIRES-2-eGFP in CA (n = 4) (**B**) and their corresponding current/pressure curves (**C**). (**D–F**) Recorded currents at 0 mV potential by applying an increased negative pressure from 0 to −80 mmHg on HEK cells transfected with kcnk2a-pIRES-2-eGFP in IO configuration (n = 7) (**D**) on HEK cells transfected with pIRES-2-eGFP in IO configuration (n = 4) (**E**) and their corresponding current/pressure curves (**F**). ^*^P value < 0.05; ^**^P value < 0.01. (**G**) Recorded currents in IO configuration by applying a voltage ramp going from −100 to +100 mV on HEK cells transfected with kcnk2a-pIRES-2-eGFP at pH 7.2 (black line) at pH 6.2 (red line) at pH 6.2 (blue line) **(**n = 9). Recorded currents on HEK cells transfected with pIRES-2eGFP at pH 5.2 (green line) (n = 3). (**H**) Typical graph showing the current amplitudes at 0 mV potential for each test conditions, ^*^P value < 0.05; ^**^P value < 0.01.

**Figure 2:**
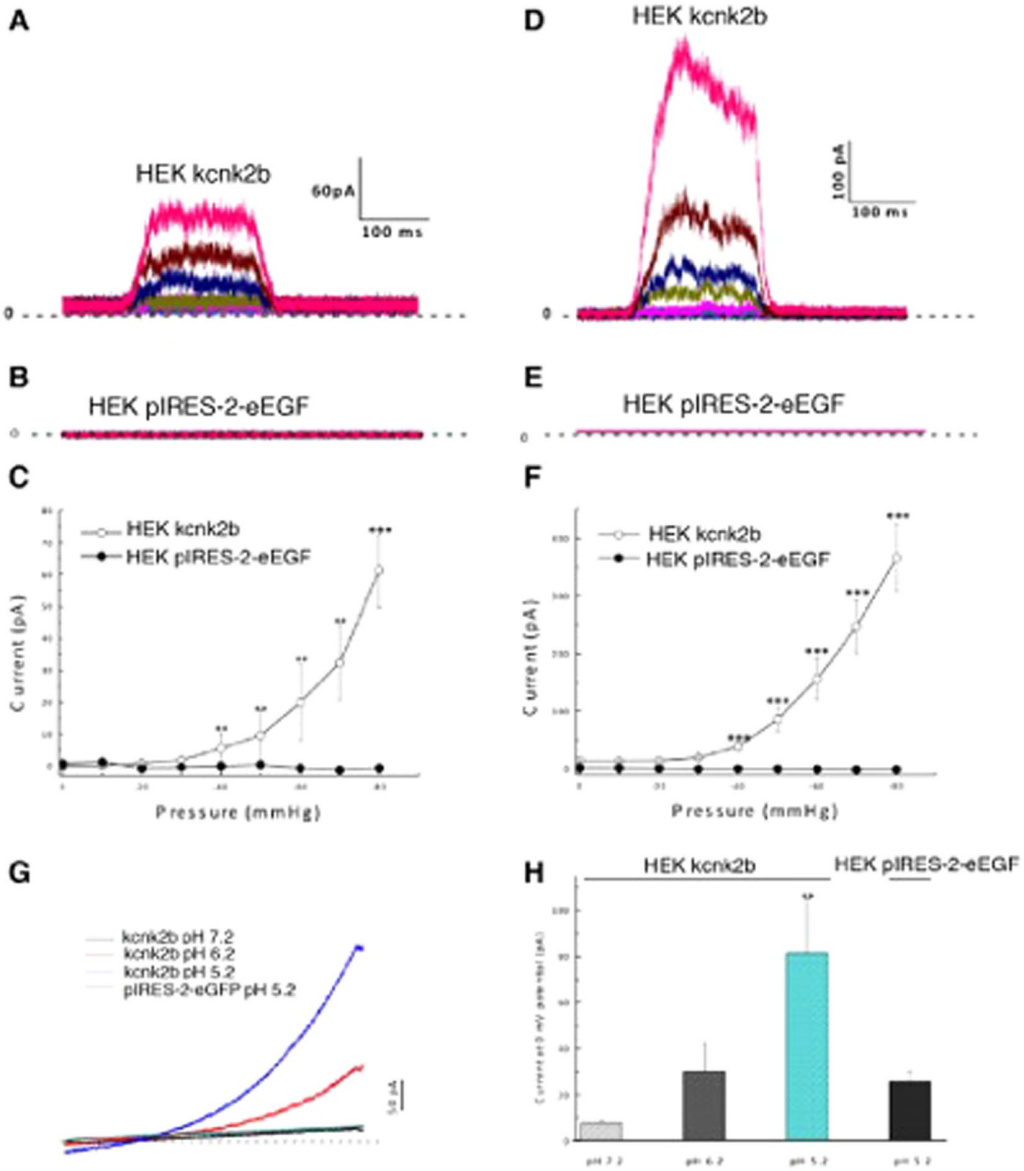
(**A–C**) Recorded currents at 0 mV potential by applying an increased negative pressure from 0 to −80 mmHg on HEK cells transfected with kcnk2b-pIRES-2-eGFP in CA configuration (n = 8) (**A**) on HEK cells transfected with pIRES-2-eGFP in CA (n = 4) (**B**) and their corresponding current/pressure curves (**C**). (**D**–**F**) Recorded currents on HEK cells transfected with kcnk2b-pIRES-2-eGFP in IO configuration (n = 19) (**D**) on HEK cells transfected with pIRES-2-eGFP in IO configuration (n = 4) (**E**) and their corresponding current/pressure curves (**F**). ^**^P value < 0.01; ^***^P value < 0.001. (**G**) Recorded currents in IO configuration by applying a voltage ramp going from −100 to +100 mV on HEK cells transfected with kcnk2b-pIRES-2-eGFP at pH 7.2 (black line) at pH 6.2 (red line) at pH 6.2 (blue line) **(**n = 11). Recorded currents on HEK cells transfected with pIRES-2eGFP at pH 5.2 (green line) **(**n = 3). (**H**) Typical graph showing the current amplitudes at 0 mV potential for each test conditions, ^**^P value < 0.01.

We next sought to determine whether both Kcnk2a and Kcnk2b responded to mechanical stretch in a similar manner to their mammalian counterparts. Both orthologs were subcloned into a pIRES2-GFP vector allowing us to express them in HEK cells for electrophysiological analysis. To ensure the consitiancy of our data, we first assessed the transfection efficiency of both constructs and found there were no significant differences associated with this procedure (Suppl. Fig. 3A–G). Using the cell attached (CA) configuration, we found that at 0 mV potential, the application of negative pressure to the cell membrane ranging from 0 to −80 mmHg resulted in an increase in current amplitude from 0 to 410.8 ± 158.3 pA for Kcnk2a (Fig. 1A,C) and 0 to 73 ± 11,4 pA for Kcnk2b (Fig. 2A,C). No current was detected from HEK cells transfected with an empty vector (Figs 1B,C and 2B,C). We repeated this analysis using an inside out (IO) configuration, and found an increase in current amplitude of 4064.6 ± 1239.4 pA at −80 mmHg for Kcnk2a (Fig. 1D,F), and 366,1 ± 59 pA at −80 mmHg for Kcnk2b (Fig. 2D,F). No current was observed from HEK cells transfected with an empty vector (Figs 1E,F and 2E,F). Our results show that Kcnk2a and Kcnk2b are mechanosentive channels which respond to membrane stretch in a similar manner to their mammalian counterparts. Interestingly, it appears that Kcnk2a is more responsive than Kcnk2b, a feature which is not caused by a difference in channel kinetics (Suppl. Fig. 4). Whether Kcnk2b can elicit physiologically relevant responses *in vivo* remains to be determined, however it is also possible that this gene has become redundant.

### Kcnk2a and Kcnk2b are activated by intracellular acidification

Previous research indicates that in mammals, KCNK2 can also be activated under acidic condition. To assess if this was also the case for either Kcnk2a or Kcnk2b, we repeated our electrophysiological analysis under decreasing pH conditions. Firstly, using the IO configuration, we lowered the pH of the perfused intracellular solution from 7.2 to 6.2 and then to 5.2. At 0 mV potential, the Kcnk2a current amplitude increased from 79.1 ± 9.3 pA at pH 7.2 to 183.6 ± 34.7 pA at pH 6.2 and to 428.8 ± 99 pA at pH 5.2 compared to the control which peaked at 25.7 ± 4.5 at pH 5.2 (Fig. 1G,H). However, for Kcnk2b the increase in current amplitudes was much more subdued ranging from 7,7 ± 1,2 pA at pH 7.2 to 29,9 ± 12,3 pA at pH 6.2 and to 81,5 ± 23,6 pA at pH 5.2 while the control reached 6,9 ± 1,3 pA at pH 5.2 (Fig. 2G,H). Taken together our data indicates that Kcnk2a and Kcnk2b both show increased activity under acidic conditions as has been reported for their mammalian counterpart. However, as we observed during the mechanical stimulation analysis, Kcnk2b appears to generate much lower current amplitudes that Kcnk2a, again indicating that these genes may be redundant.

### Kcnk2a and Kcnk2b can be activated by the polyunsaturated fatty acids DHA and AA

**Figure 3:**
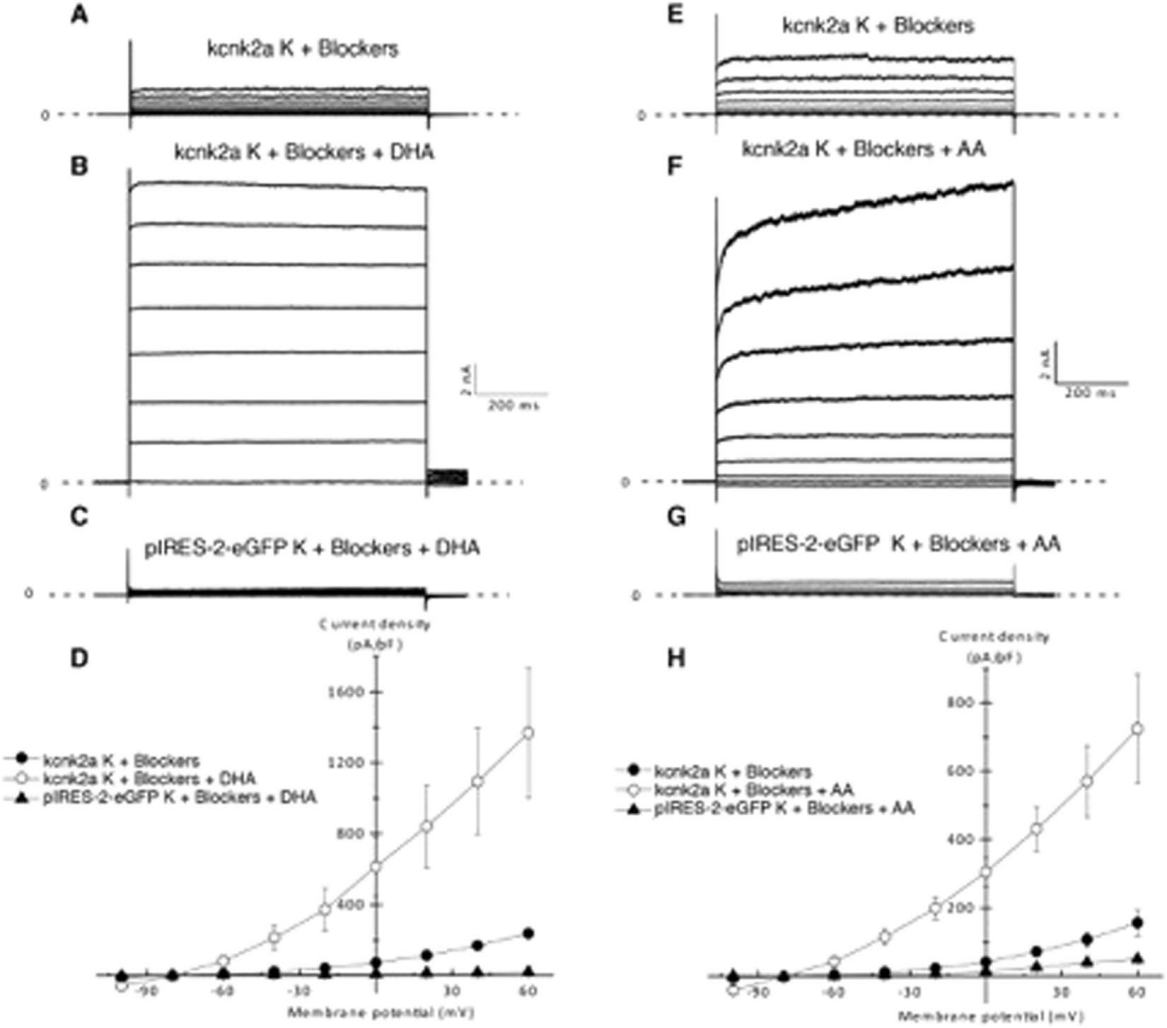
(**A,B,E,F**) Recorded currents in WC configuration by applying a voltage steps protocole going from −100 to + 60 mV by – 20 mV increments. on HEK cells transfected with kcnk2a-pIRES-2-eGFP in the presence of potassium cocktail blockers (**A,E**) (n = 5), in the presence of potassium cocktail blockers and unsaturated fatty acid 10 µM of Docosahexaenoic acid (DHA) (n = 5) (**B**) or 10 µM of arachidonic acid (AA) (n = 5) (**F**). (**C**,**G**) Recorded currents on HEK cells transfected pIRES-2-eGFP in presence of the potassium cocktail blockers and 10 µM of each polyunsaturated fatty acid DHA (n = 5) **(C)** or AA (n = 5) (**G**). (**D**,**H**) Corresponding Current/potential curves for each tested condition.

**Figure 4:**
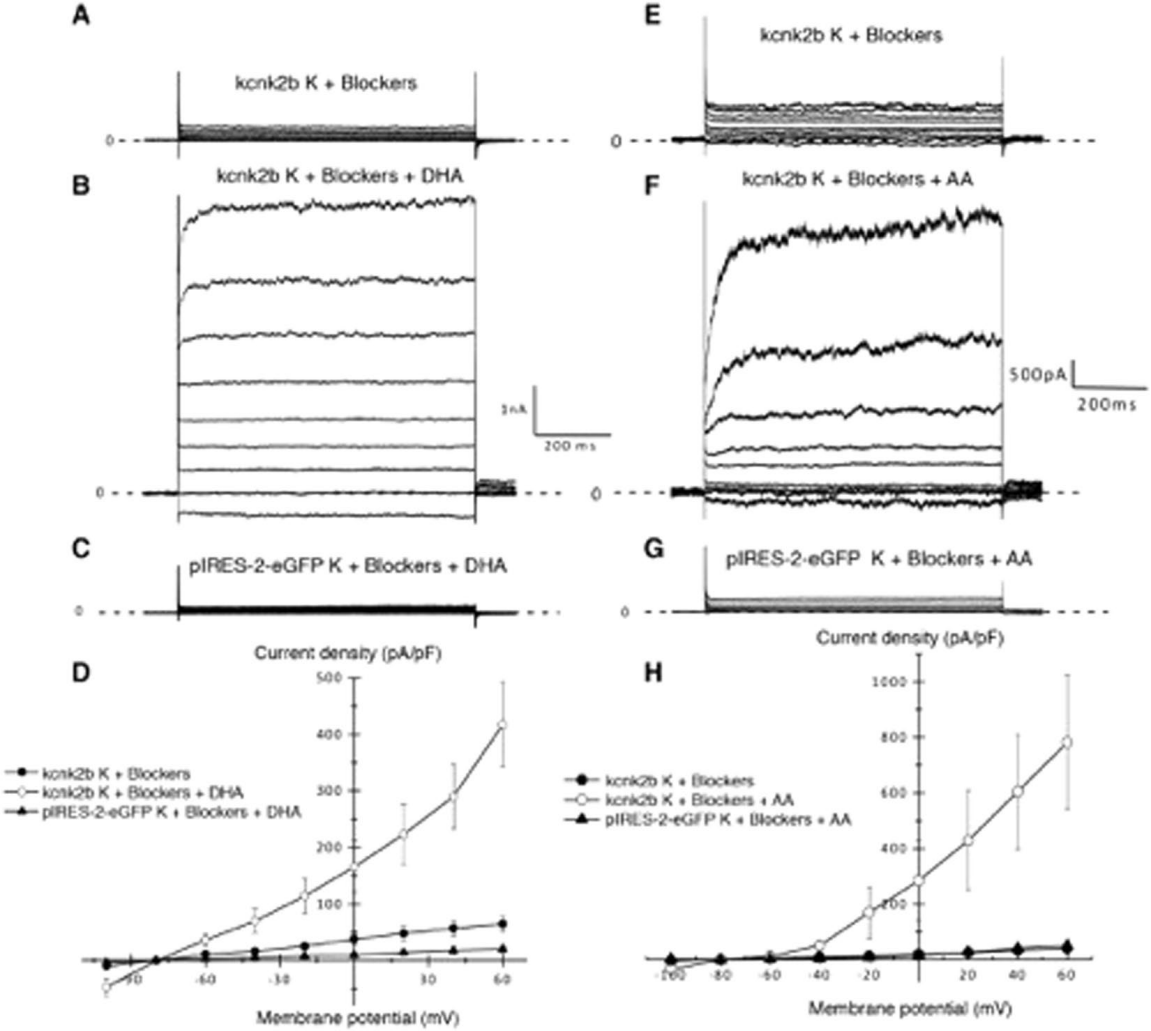
(**A,B,E,F**) Recorded currents in WC configuration by applying a voltage steps protocole going from −100 to +60 mV by −20 mV increments on HEK cells transfected with kcnk2b-pIRES-2-eGFP in the presence of potassium cocktail blockers (n = 5) (**A**,**E**) in the presence of potassium cocktail blockers and unsaturated fatty acid 10 µM of Docosahexaenoic acid (DHA) (n = 5) (**B**) or 10 µM of arachidonic acid (AA) (n = 5) (**F**). (**C**,**G**) Recorded currents on HEK cells transfected pIRES-2-eGFP in presence of the potassium cocktail blockers and 10 µM of each polyunsaturated fatty acid DHA (n = 5) (**C**) or AA (n = 5) (**G**). (**D**,**H**) Corresponding Current/potential curves for each tested condition.

We next sought to evaluate if PUFAs such as DHA and AA are able to activate either Kcnk2a or Kcnk2b in a similar manner as has been observed for mammalian Kcnk2. Basal Kcnk2a currents were recorded in the whole cell (WC) configuration using HEK cells transfected with Kcnk2a-pIRES-2-eGFP in the presence of a cocktail of potassium channel inhibitors (4-AP, TEA, glibenclamide, apamin, charybdotoxin) (Fig. 3A,D). Currents are presented as current densities pA/pF. Perfusion of DHA 10 µM promotes an increase of current density amplitudes from 72,52 ± 3,48 pA/pF to 615,10 ± 166,87 at 0 mV potential (Fig. 3B,D). In the presence of K+ blockers cocktail DHA perfusion on HEK cells transfected with pIRES-2-eGFP empty vector does not promote an increase of basal current amplitude (Fig. 3C). For Kcnk2b we found that the same treatment with DHA from kcnk2b basal current (Fig. 4A) resulted in an increase of current density amplitudes from 37,3 ± 9,8 pA/pF to 165,3 ± 45,1 at 0 mV potential on (Fig. 4B,D). In the presence of K+ blockers cocktail DHA perfusion on HEK cells transfected with pIRES-2-eGFP empty vector does not promote an increase of basal current amplitude (Fig. 4C). Next we repeated these assays substituting DHA for AA. In this manner we found that perfusion of 10 µM of AA from basal kcnk2a and kcnk2b currents (Figs 3E, 4E) promoted an increase of current density amplitudes from 42,35 ± 8,81 to 304,70 ± 41,96 pA/pF at 0 mV potential for Kcnk2a (Fig. 3F,H) and 19,7 ± 7,6 to 284,7 ± 143,6 for Kcnk2b (Fig. 4F,H). In the presence of K+ blockers cocktail AA perfusion on HEK cells transfected with pIRES-2-eGFP empty vector does not promote an increase of basal current amplitude (Fig. 3G, 4G).These data indicate that both Kcnk2a and Kcnk2b are positively regulated by the PUFAs DHA and AA as has been reported for the mammalian counterpart.

### Spadin, a KCNK2 inhibitory peptide, also blocks Kcnk2a and Kcnk2b activity

**Figure 5:**
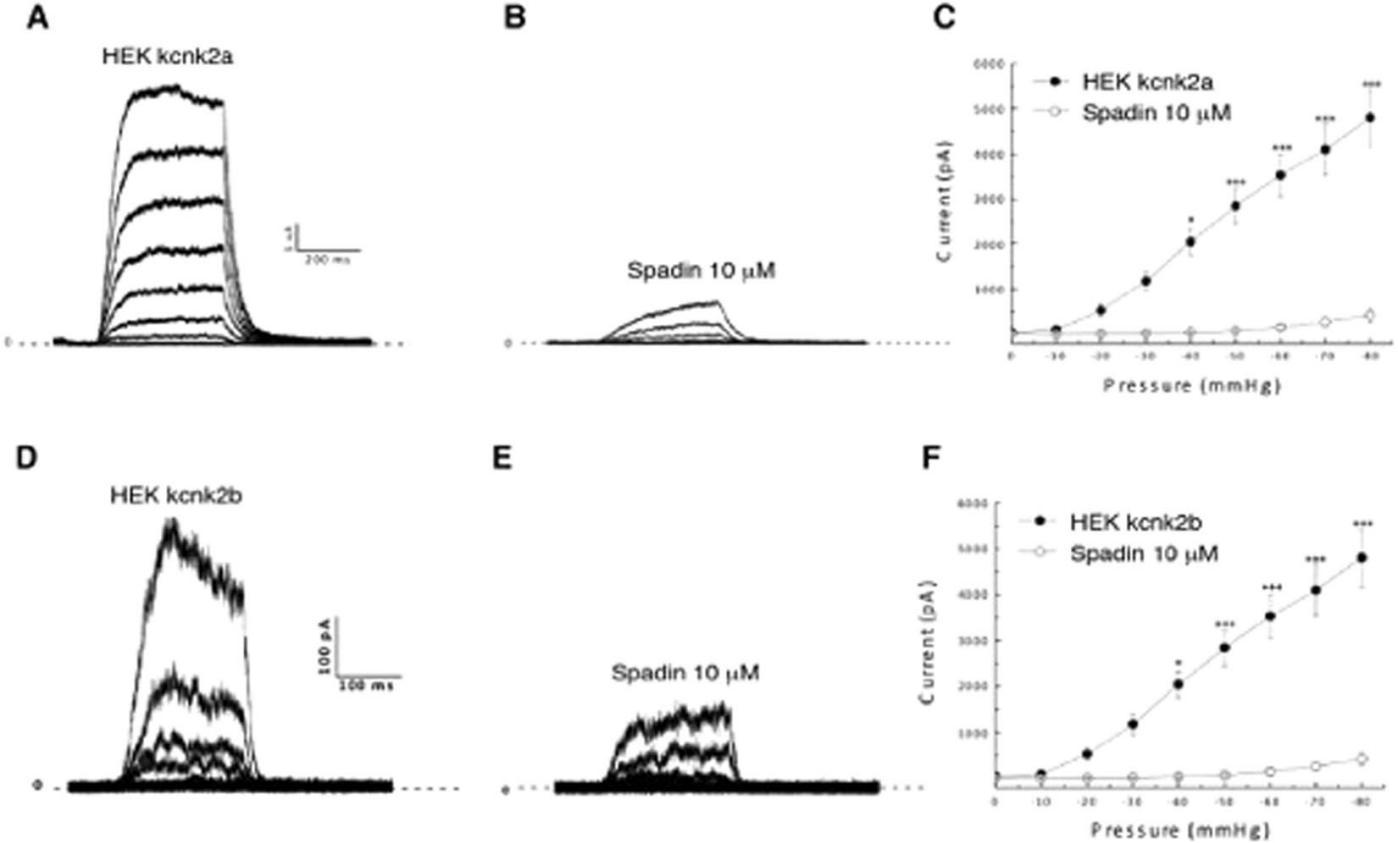
Recorded currents in IO configuration by applying an increasing negative pressure protocol from 0 to −80 mmHg by – 10 mmHG pressure step increments on HEK cells transfected with kcnk2a-pIRES-2-eGFP (control condition) (n = 7) (**A**) in the presence of 10 µM of spadin into the pipette medium (n = 6) (**B**) and their corresponding current/pressure curves (**C**). Recorded currents in IO configuration by applying an increasing negative pressure protocol from 0 to −80 mmHg by – 10 mmHG pressure step increments on HEK cells transfected with kcnk2b-pIRES-2-eGFP (control condition) (n = 7) (**D**) in the presence of 10 µM of spadin into the pipette medium (n = 6) (**E**) and their corresponding current/pressure curves (**F**). ^*^P value < 0.05, ^***^P value < 0.001.

In order to evaluate the effects of Spadin, a KCNK2 channel inhibitor, on Kcnk2a and Kcnk2b, we repeated the mechanostimulation protocol in the presence or absence of this molecule. Typical Kcnk2a currents were recorded in control conditions with an amplitude of 3526 ± 482,4 pA at −60 mmHG pressure stimulation (Fig. 5A,C). Following the introduction of 10 µM of spadin into the pipette medium, we observed a significant decrease of the recorded current from 3526 ± 482,4 to 148,4 ± 56,5 pA at – 60 mmHG (Fig. 5B,C). Likewise we also observed that Spadin produced a significant inhibitory effect on Kcnk2b by reducing the control current amplitude (Fig. 5D,F) from 156,3 ± 36,5 to 42 ± 9,2 pA at – 60 mmHG (Fig. 5E,F). Furthermore, spadin inhibition only affected the current amplitude and did not disrupt the channel kinetics (Suppl. Fig. 4). Lastly, we assessed whether spadin was capable of inhibiting kcnk2 currents in zebrafish cardiomyocytes. To achieve this we first established primary cultures of adult zebrafish cardiomyocytes and performed electrophysiological recordings from individual cardiomyocytes. In this manner we were able to detect Kcnk2 like currents following negative pressure simulation (Suppl. Fig. 5A,C). Importantly these currents could be significantly inhibited when the cardiomyocytes were treated with spadin, indicating that a Kcnk2 must be responsible for significant proportion of the measured current observed in zebrafish cardiomyocytes (Suppl. Fig. 5B,C).These data indicate that spadin, an inhibitory peptide developed to target KCNK2, significantly inhibits both Kcnk2a and Kcnk2b. This also indicates that the zebrafish orthologs of KCNK2 will be useful for developing targeting strategies for this gene.

## Discussion

Here we have identified two zebrafish orthologs of *KCNK*, namely *kcnk2a* and *kcnk2b*. Both orthologs are highly homologous to their human counterpart including many known KCNK2 regulatory residues. Furthermore, both orthologs are also highly expressed in the developing zebrafish brain as has been reported in mammals and humans. We were also able to detect Kcnk2 channel currents in zebrafish cardiomyocytes, however we were not able to determine whether these currents were generated solely by one of the orthologs or, are a combination of the the two. Our electrophysiological recordings indicate that both channels are sensitive to mechanostimulation as has been observed for the mammalian KCNK^8^. Indeed, the increase in current amplitude observed between cell attached and inside out configurations indicates that both Kcnk2a and Kcnk2b are also negatively regulated by the cytoskeleton in response to mechanostimulation^8,9^. KCNK2 also acts as a cytoplasmic pH sensor whereby increasing intracellular acidity induces channel activation^8,9^. This particular property suggests a putative protective role for KCNK2 against ischemia and pain, in which disrupted metabolism promotes intracellular acidification^29,42–44^. Consequently, the increasing acidity triggers KCNK2 to open resulting in cell membrane hyperpolarisation, a protective phenomenon also produced by ATP sensitive potassium channels during cardiac and cerebral ischemia^45,46^. Here we also demonstrate that both Kcnk2a and Kcnk2b respond positively when the intracellular pH decreases from 7.2 to 5.2. PUFAs represent another mode by which KCNK2 can be activated. Indeed, arachidonic acid (AA), docosahexaenoic acid (DHA), alpha linolenic acid ALA, ecosapentaenoic acid (EPA), lysophospholipids (LP) such as lysophosphatidylcholine (LPC) have all been shown to be capable of inducing KCNK2 activity^9,11–14^. Preventative therapies utilising PUFAs have been developed to tackle cardiovascular and cerebrovascular diseases such as myocardial infarction, cerebral stroke, pain and epillepsia. One of the ways in which PUFAs produce a protective effect is *via* potassium channel mediated cell membrane hyperpolarization, which in turn promotes cell survival under pathological conditions. For example, cell membrane hyperpolarization promotes a significant decrease in stroke damage in mouse models of cerebral focal ischemia^12,13,29,42,47^. These results are supported by *in vitro* data showing the protective role PUFA mediated KCNK2 activation has on primary cultures of neurons during oxygen/glucose deprivation studies^9,47^. Here we have shown that both zebrafish *KCNK2* orthologs are also activated by the PUFAs DHA and AA in a similar manner to their mammalian counterparts indicating that zebrafish represent a potentially useful *in vivo* model for further development of PUFAs based therapeutic strategies. Although both Kcnk2a and Kcnk2b show similar biophysical properties and polymodal activation to mammalian KCNK2 channels, we were able to determine a very obvious difference in active current amplitudes between the two zebrafish orthologs. The presence of two copies of the same gene in zebrafish is not uncommon and has been attributed to an ancient whole genome duplication event. Our data indicates that Kcnk2b appears to generate much lower current amplitudes than Kcnk2a following different modes of activation (although there appears to be no difference in the channel kinetics). One explanation for this difference is that over time Kcnk2b has become redundant to Kcnk2a, however more extensive research, for example generation of CRISPR/Cas9 knockout zebrafish lines, will be required to determine whether Kcnk2b can compensate for Kcnk2a and *vice versa*. Furthermore, a detailed comparison of the composition/structure between Kcnk2a and Kcnk2b may provide valuable information about how these channels function. Depression in humans can be treated by selective serotonin reuptake inhibitors (SSRIs) such as fluoxetine and norefluoxetine^15,17,48^. Both these compounds have been shown to effectively inhibit KCNK2 which has made this channel an attractive pharmacological target to treat depression^2,15^. Indeed, over the last decade, several molecules have been developed to specifically inhibit KCNK2 activity such as the short peptide Spadin^9,16,20–24^. This molecule has been shown to display a therapeutic effect in mouse models of depression, a feature linked to its specific inhibition KCNK2. Our data indicates that Spadin also effectively inhibits both Kcnk2a and Kcnk2b following mechanostimulation indicating that there is sufficient homology between the zebrafish and mammalian channels for testing KCNK2 specific pharmacology. Taken together, our data lays the groundwork for developing the zebrafish as an *in vivo* model for KCNK2 targeted therapeutic strategies.

## Methods

### Kcnk2a and Kcnk2b cloning

*Kcnk2a* and *kcnk2b* were cloned from a 3 days post fertilisation zebrafish cDNA library using the following nested sets of primers:

### kcnk2a

Forward primer 5′ AGCGAGAACAGCAGATCCCA 3′

Reverse primer 5′ GCTTACATTTTAGTATGTGC 3′

Forward nested primer 5′ ATGGCTGCACCTGATCTTTT 3′

Reverse nested primer 5′ TTATTTGAGATGTTCAATGA 3′

### kcnk2b

Forward primer 5′ GCTGCTGAAGCCTCCAGAGG 3′

Reverse primer 5′ CAGCTTGTCCTTTGAATTTC 3′

Forward nested primer 5′ ATGCGCTGGAAGACCGTGCT 3′

Reverse nested primer 5′ TCATTTTGTCTGTATTCTAG 3′.

PCR products were initially cloned into pGEMT Easy and sequenced before subcloning into pIRES2-GFP.

### *In situ* hybridisation

Anti-sense probes were synthesized as described previously^49^. *In situ* hybridization were performed as described previously^50^.

#### Cell culture and transfection

Both channels were expressed in HEK-293 cells (American type culture collection, Manassas, VA, USA) by transient transfection using JetPei cationic lipids (Ozyme, France) following the manufacturers protocol. HEK-293 cells were grown in Dulbecco’s modified Eagle’s medium (DMEM) supplemented with 10% (v/v) heat inactivated fetal bovine serum (FBS) and 1% (v/v) penicillin/streptomycin and 95% air/5% CO_2_. HEK-293 cells were plated in 35 mm diameter dishes for transfection at a density of 15 000 cells per dish. 0.5–1 µg of each plasmid was used to transfect 10 dishes of HEK-293 cells. Transfected cells were assayed two days after transfection and during the three following days. Transfected cells were maintained in DMEM culture medium (10% FBS, 1% penicillin/streptomycin) in 95% air/5% CO_2_.

### Cardiomyocyte isolation and culture

Adult zebrafish cardiomyocytes were isolated and cultured as described previously^51^.

#### Electrophysiology

Whole cell current recordings: Each current was calculated by using an axopatch 200B amplifier (Axon Instrument, Sunnyvale, CA, USA), low-pass filtered 3 kHz and digitized at 10 kHz using a 12-bits analog to digital converter digidata (1440 A series, Axon Instrument, Sunnyvale, CA, USA). Patch clamp pipettes were pulled using vertical puller PC-10 (Narashighe, London, UK) from borosilicate glass capillaries with a resistance between 3–5 MΩ. The bath solution contained (in mM) 150 NaCl, 5 KCl, 3 MgCl_2_, 1 CaCl_2_ and 10 HEPES adjusted to pH 7.4 with NaOH. The pipette solution contained (in mM) 155 KCl, 3 MgCl_2_, 5 EGTA and 10 HEPES adjusted to pH 7.2 with KOH. All experiments were performed at room temperature (22 °C). Kcnk2a and Kcnk2b currents were measured in the presence of a cocktail of potassium channel inhibitors (K+ blockers: 3 mM 4-aminopyridine (4-AP), 10 mM tetraethylamonium (TEA), 10 µM glibenclamide, 100 nM apamin and 50 nM charybdotoxin). Stimulation protocols and data acquisition were carried out using a microcomputer (Dell pentium), which used commercial software and hardware (pClamp 10.2). Cells were clamped at −80 mV and voltage changes were applied by steps of 20 mV from −100 to +60. Duration of depolarization pulses was 0.825 ms and the pulse cycling rate was 5 s. Kcnk2a and Kcnk2b current amplitudes were calculated at the end of the stimulation pulses. Cells were continousely perfused with a microsuperfusion system. Electrophysiological and pharmacological Kcnk2a and Kcnk2b current characterization was obtained using two KCNK2 activators, arachidonic acid AA and docosaexaenoic acid DHA. One concentration at 10 µM was used to perfuse the cells with these two PUFAs. Current amplitudes were expressed in current densities. Results are expressed as mean ± standard error of the mean (SEM).

### Mechanical activation by cell membrane stretch

Kcnk2a and Kcnk2b mechanical activation by cell membrane stretch was performed in both cell attached (CA) and inside out (IO) configurations. The bath solution contained (in mM) 155 KCl, 3 MgCl_2_, 5 EGTA and 10 HEPES adjusted to pH 7.2 with KOH. The pipette solution contained (in mM) 150 NaCl, 5 KCl, 2 CaCl_2_ and 10 HEPES adjusted to pH 7.4 with NaOH. Patch pipettes were around 1.2–1.5 MΩ and cell membranes were stimulated with negative pressure pulses from 0 to −80 mmHG in −10 mmHG increments during 300 ms each 3 s, through the recording electrode using a pressure clamp device (ALA High Speed Pressure Clamp-1 system, ALA-scientific).

### Activation by intracellular acidification

Kcnk2a and Kcnk2b activation by intracellular acidification was performed in inside out (IO) configuration. Cells were stimulated by a voltage ramp protocol from −120 mV to 60 mV during 550 ms each 3 s. Holding potential was maintained at −80 mV. Cell membranes were continuously perfused by the pH 7.2 control solution, and by both experimental pH 6.2 and 5.2 solutions until a steady state was achieved. The bath and pipette mediums for the pH experiments were the same inside out mediums described in the mechanical activation method. For all experiments, currents were filtered at 1 KHz, digitized at 20 kHz and analyzed with pClamp 10.2 and Origin 8.0 softwares. Data is expressed as mean ± S.E.M. Statistical analysis of differences between groups was performed using a students unpaired t test. In all analyses, the level of significance is (*)p < 0.05, (**)P < 0.01 and (***)P < 0.001.

## Electronic supplementary material

Supplementary figures
