# Supplementary material for: Identification and characterization of two zebrafish Twik related potassium channels, Kcnk2a and Kcnk2b
Source: Sci Rep. 2018 Oct 17;8:15311. doi: 10.1038/s41598-018-33664-9 (PMC6192994; doi:10.1038/s41598-018-33664-9)
Supplement: Supplementary file 1 — Supplementary figures [file 41598_2018_33664_MOESM1_ESM.pdf]

Identification and characterization of two zebrafish Twik related potassium channels, Kcnk2a and Kcnk2b.

Nathalie Nasr[1], Adèle Faucherre[1], Marc Borsotto[2], Catherine Heurteaux[2], Jean Mazella[2], [*]Chris Jopling[1], [*]Hamid Moha ou Maati[1]

```
hTREK1    MLPSASRERPGYRAGVAAPDLLDPKSAAQNSKPRLSFSTKPTVLASRVESDTTINVMKWK    60
mTREK1    MLASASRERPGYTAGVAAPDLLDPKSAAQNSKPRLSFSSKPTVLASRVESDSAINVMKWK    60
Kcnk2a    --MRSSENSRSHKVVMAAPDLLDPKSATHNTKPRLSFSSKPIVYNSGDDCESITTVMKWK    58
Kcnk2b    --------------------------------------------------------MRWK    4
                                                                  *:**

hTREK1    TVSTIFLVVVLYLIIGATVFKALEQPHEISQRTTIVIQKQTFISQHSCVNSTELDELIQQ    120
mTREK1    TVSTIFLVVVLYLIIGATVFKALEQPQEISQRTTIVIQKQTFIAQHACVNSTELDELIQQ    120
Kcnk2a    TVLAIFLLVVLYLIIGATVFKALEQPEEGLQKYRIIQEKIDFLSMHTCVNTSELEDLVKQ    118
Kcnk2b    TVLSVFLLVVLYLILGATVFKELEQPYETLQKLNILMEKLEFLEQHPCVNSSDLENLVKQ    64
          ** ::**:******:****** **** *  *:  *: :*  *:  * ***:::*::*::*

hTREK1    IVAAINAGIIPLGNTSNQISHWDLGSSFFFAGTVITTIGFGNISPRTEGGKIFCIIYALL    180
mTREK1    IVAAINAGIIPLGNSSNQVSHWDLGSSFFFAGTVITTIGFGNISPRTEGGKIFCIIYALL    180
Kcnk2a    VVLAIRAGVNPSGHPSNESSMWDLSSSFFFAGTVITTIGFGNVSPHTEGGRIFCIIYALL    178
Kcnk2b    VVSALRAGVNPSGNSSNQSSLWDLSNSFFFSGTVITTIGFGNISPHTEVGRIFCIIYALL    124
          :* *:.**: * *: **: * ***..****:***********:**:** *:*********

hTREK1    GIPLFGFLLAGVGDQLGTIFGKGIAKVEDTFIKWNVSQTKIRIISTIIFILFGCVLFVAL    240
mTREK1    GIPLFGFLLAGVGDQLGTIFGKGIAKVEDTFIKWNVSQTKIRIISTIIFILFGCVLFVAL    240
Kcnk2a    GIPLFGFLLAGVGDQLGTIFGKGIAKVEKMFVKWNVSQTKIRVTSTVLFILFGCLLFVAL    238
Kcnk2b    GIPLFGFLLAGVGDQLGTIFGKAIAKVEGMIDKWNVSQTKIRVISTLLFILFGCLLFVTL    184
          **********************.*****  : **********: **::******:***:*

hTREK1    PAIIFKHIEGWSALDAIYFVVITLTTIGFGDYVAG------------GSDIEYLDFYKPV    288
mTREK1    PAVIFKHIEGWSALDAIYFVVITLTTIGFGDYVAG------------GSDIEYLDFYKPV    288
Kcnk2a    PALIFQHIEGWSALESIYFVVITLTTIGFGDFVAG------------GSEIEYLDYYKPI    286
Kcnk2b    PAVIFKHIEGWSALESIYFVVITLTTIGFGDFVAGEAERRHHEESSGGSQLEYLDYYKPL    244
          **:**:********::***************:***            **::****:***:

hTREK1    VWFWILVGLAYFAAVLSMIGDWLRVISKKTKEEVGEFRAHAAEWTANVTAEFKETRRRLS    348
mTREK1    VWFWILVGLAYFAAVLSMIGDWLRVISKKTKEEVGEFRAHAAEWTANVTAEFKETRRRLS    348
Kcnk2a    VWFWILVGLAYFAAVLSMIGDWLRVISKKTKEEVGEFRAHAAEWTANVSAEFKETRRRLS    346
Kcnk2b    VWFWILVGLAYFAAVLSMIGDWFRVISKKTKEEVGEFRAHAAEWTANVSAEFKETRRRLS    304
          **********************:*************************:***********

hTREK1    VEIYDKFQRATSIKRKLSAELAGNH--NQELTPCRRTLSVNHLTSER-DVLPPLLKTESI    405
mTREK1    VEIYDKFQRATSVKRKLSAELAGNH--NQELTPCRRTLSVNHLTSER-EVLPPLLKAESI    405
Kcnk2a    VEIYDKFQRAAYIKRKLSSELGQNP--GQDMMPCKRTLSVNFTDELEKEGLPTLTKNGSL    404
Kcnk2b    VDIYDKFQRATSVKRKLSAEINLSPPINQQMTPGKRARSVNLGDEREAY-PYTLARNGSL    363
          *:********: :*****:*:  .   .*:: * :*: ***   . .      * :  *:

hTREK1    YLNGLTPHCAGEE-IAVIENIK    426
mTREK1    YLNGLTPHCAGED-IAVIENMK    426
Kcnk2a    YLNGLTPDCPDCSEISIIEHLK    426
Kcnk2b    FLNSLIPDYADHRDMTRIQTK-    384
          :**.* *.  .   :: *:
```

Supplementary Figure 1: Protein alignments of both Kcnk2a and Kcnk2b with the human and mouse KCNK2 channels

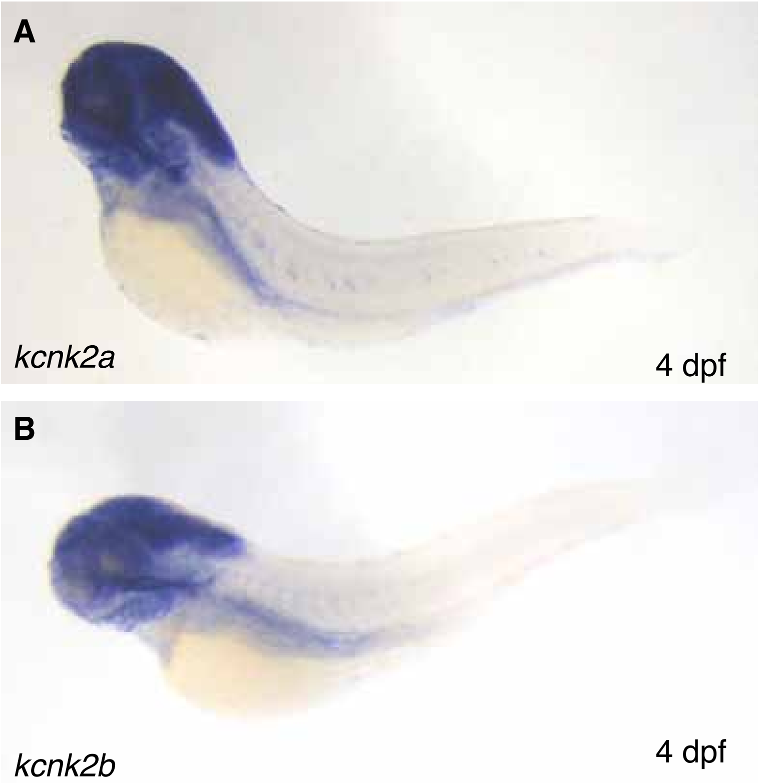

Supplementary Figure 2: *In situ* hybridization on wild type zebrafish larvae at 4 days post fertilization (4 dpf) using an antisense probe against *kcnk2a* **(A)** or *kcnk2b* **(B).**

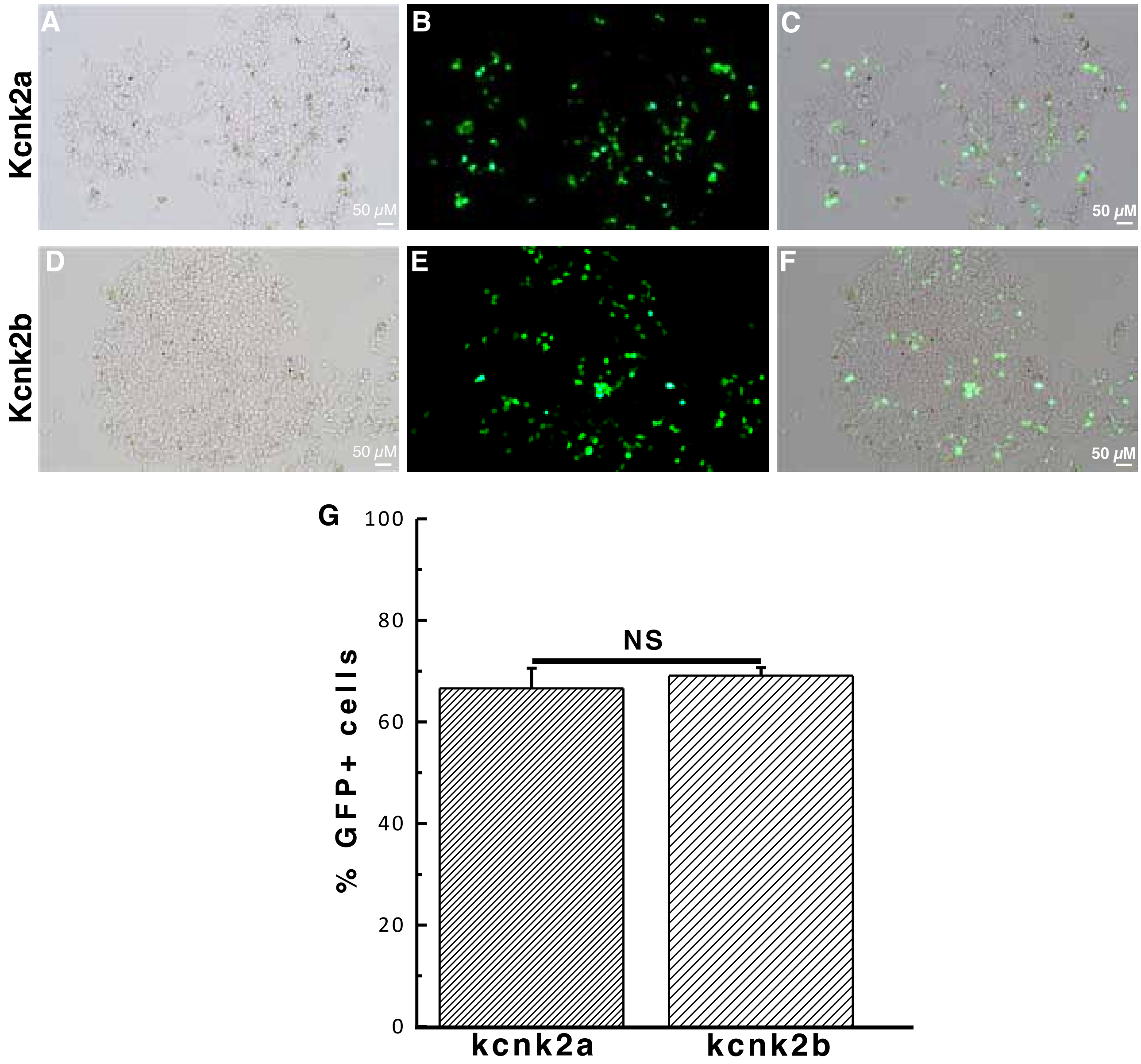

Supplementary Figure 3: Kcnk2a and kcnk2b transfection efficiency.

(A) Brightfield image of HEK-293T transfected with *kcnk2a*-PIRES2 GFP. **(B)** GFP fluorescence image of HEK-293T transfected with *kcnk2a*-PIRES2 GFP. **(C)** Merged image of A and B. (**D**) Brightfield image of HEK-293T transfected with *kcnk2b*-PIRES2 GFP. **(E)** GFP fluorescence image of HEK-293T transfected with *kcnk2b*-PIRES2 GFP. **(F)** Merged image of D and E. **(G)** Graph showing the overall transfection efficiency of HEK-293T with either *kcnk2a*-PIRES2 GFP or *kcnk2a*-PIRES2 GFP. 8x35mm dishes were plated with 3×10^5 HEK-293T cells and transfected with either *kcnk2a*-PIRES2 GFP (4 dishes) or *kcnk2a*-PIRES2 GFP (4 dishes). 5 non overlapping random wide field images were obtained and the mean transfection efficiency was calculated for each dish. The data in (G) is the average of the combined means. Statistics: Students t test, NS (not significant) p>0,05.

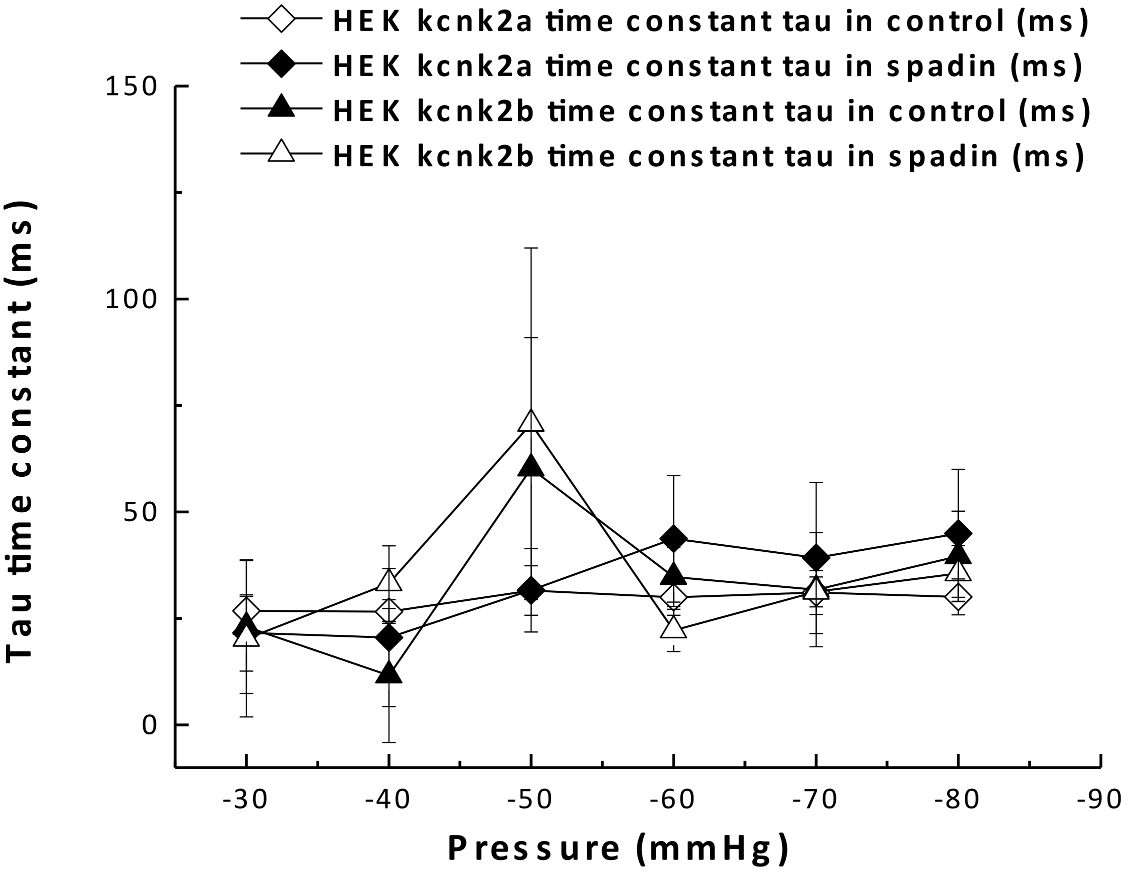

Supplementary Figure 4: Comparison of Kcnk2a and Kcnk2b channel kinetics.

Graph depicting the calculated τ (tau) time constant ($f(t) = \sum_{i=1}^{n} A_i e^{-t/\tau_i} + C$) for Kcnk2a and Kcnk2b transfected HEK-293T cells in control conditions and in the presence of spadin (n= 6 cells per point). Statistics: students t test comparing Kcnk2a and Kcnk2b in control conditions and in the presence of spadin. NS (not significant) p>0,05.

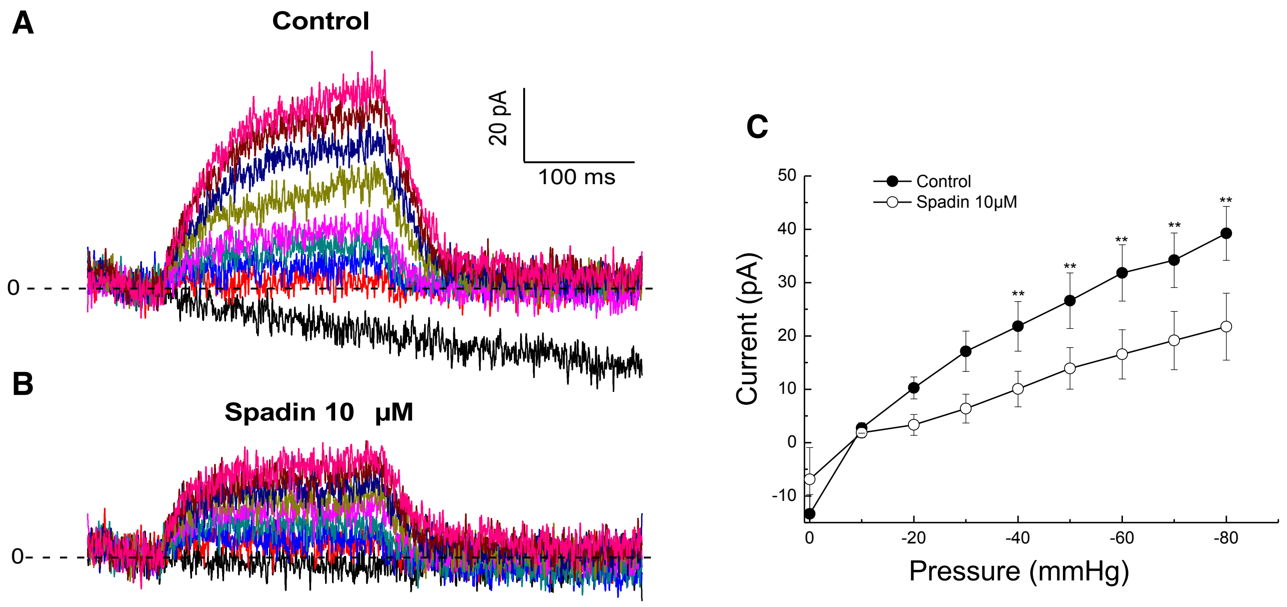

Supplementary Figure 5: Detection of Kcnk2 channel currents in zebrafish cardiomyocytes.

(A) Typical current trace recorded in control conditions, at 0 mv potential in the cell attached configuration with a negative pressure protocol from 0 to -80 mmHg in -10 mmHg step increments. **(B)** Typical current trace recorded in the presence of spadin, under the same conditions. **(C)** Current pressure curves showing the amplitude of the current for each pressure step in the absence (control) and presence of spadin (Spadin). Statistics: students t test comparing each pressure step in control and spadin conditions, ** p<0,01.
